# Metabolomic Analysis and MRM Verification of Coarse and Fine Skin Tissues of Liaoning Cashmere Goat

**DOI:** 10.3390/molecules27175483

**Published:** 2022-08-26

**Authors:** Yanan Xu, Weidong Cai, Rui Chen, Xinjiang Zhang, Zhixian Bai, Yu Zhang, Yuting Qin, Ming Gu, Yinggang Sun, Yanzhi Wu, Zeying Wang

**Affiliations:** College of Animal Science &Veterinary Medicine, Shenyang Agricultural University, Shenyang 110866, China

**Keywords:** cashmere fineness, metabolomics, Liaoning cashmere goat, UHPLC–MS/MS, MRM

## Abstract

One of the critical elements in evaluating the quality of cashmere is its fineness, but we still know little about how it is regulated at the metabolic level. In this paper, we use UHPLC–MS/MS detection and analysis technology to compare the difference in metabolites between coarse cashmere (CT_LCG) and fine cashmere (FT_LCG) skin of Liaoning cashmere goats. According to the data, under positive mode four metabolites were significantly up-regulated and seven were significantly down-regulated. In negative mode, seven metabolites were significantly up-regulated and fourteen metabolites were significantly down-regulated. The two groups’ most significant metabolites, Gly–Phe and taurochenodeoxycholate, may be crucial in controlling cashmere’s growth, development, and fineness. In addition, we enriched six KEGG pathways, of which cholesterol metabolism, primary bile acid biosynthesis, and bile secretion were enriched in positive and negative modes. These findings offer a new research idea for further study into the critical elements influencing cashmere’s fineness.

## 1. Introduction

Cashmere is one of the most popular raw materials on the market, which can be used to produce cashmere textiles that keep out the cold in people’s daily life. The Liaoning cashmere goat (LCG) is a local breed in southeastern Liaoning Province, China, and its cashmere yield is very high worldwide. However, the growth of cashmere is affected by many factors, such as climate, variety, sex, gene, and nutrient absorption [1]. In recent years, more and more people have begun to pay attention to the influence of the characteristics of cashmere fiber on the quality of cashmere, and the fineness of cashmere is one of the critical factors affecting the quality of cashmere [2]. Despite the excellent cashmere yield of LCG, it has a rough fineness. Therefore, it is urgent to find new research methods to reduce the fineness of cashmere.

Metabolomics is a powerful tool for understanding the overall changes in metabolic response and studying the phenotype of objects through instrumental analysis, and it can more accurately reflect the physiological state of organisms [3,4]. As metabolites are the final products of cellular activity, their concentrations may be seen as the biological systems’ final response to genetic or environmental changes [4]. By metabolomic analysis of maternal hair during pregnancy, Delplancke et al. found significant differences in some hair metabolites in early and late gestation and in healthy individuals and diabetics in late gestation. This research illustrates that variation in the amounts of amino acids, TCA cycle intermediates, fatty acids, cofactors, vitamin-related metabolites, and xenobiotics can be reflected in alterations in the hair metabolome during pregnancy [5]. Wang et al. reported that the skin of rats in the chronic restraint stress (CRS) group showed significant modifications in the primary and secondary metabolic pathways of carbohydrate metabolism, amino acid metabolism, and lipid metabolism through the metabonomic analysis of hair growth induced by CRS in rats [6]. Each functional state of the skin has its specific metabolite pattern, which can also reflect the underlying cells and mechanisms of action [7]. By mass spectrometry analysis of skin and blood samples from patients with psoriasis, Dutkiewicz et al. found that metabolites on diseased skin, especially choline and citrulline, showed more significant dynamics [8]. Pohla et al. compared the metabonomic characteristics of lesional and non-lesional skin between patients with plaque psoriasis and healthy controls. The results revealed substantial alterations in the concentration levels of 29 metabolites, indicating that the primary driver of metabonomic changes was the local inflammatory process promoting cell proliferation [9].

Currently, the research on metabolomics in goats is mainly focused on milk. Caboni et al. used gas chromatography coupled with mass spectrometry untargeted metabolomics approach to study the metabolites of 30 kinds of sheep milk and 28 kinds of goat milk. The finding demonstrated a relationship between the concentration of metabolites and the protein and fat levels in sheep and goat milk, which played a role in better understanding milk metabolism and evaluating milk properties [10]. Goat milk samples from three different pastures and three different lactation phases were examined by Liu et al. using the untargeted metabolomics technique. According to the findings, 2 types of lipids and 26 types of lipids in milk at various lactation phases were shown to differ significantly. There are a total of 38 and 19 lipid molecules that may be employed as possible markers for determining a region’s origin and lactation stage, respectively [11]. Using a combination of multivariate statistical data processing and gas chromatography–mass spectrometry, Paola Scano et al. found that the type of milk can be distinguished according to the polar metabolite spectrum of milk, which can more easily detect food fraud and protect the uniqueness of goat milk [12]. Nuclear magnetic resonance technology was used by Sun et al. to study the metabolic spectrum of Hu sheep during healthy pregnancy. They discovered that related metabolites are crucial for amino acid and lipid metabolism during normal pregnancy to meet the nutritional needs of pregnant ewes [13]. Although there are no articles on studying the fineness of cashmere through metabolomics, there are functional metabolites such as amino acids and lipids in hair [14]. According to the pertinent research ideas of others, it is possible to use metabolomics to screen the essential metabolites determining cashmere fineness.

In this work, the metabolites of the Liaoning cashmere goat’s coarse cashmere (CT_LCG) and fine cashmere (FT_LCG) skin were examined using the UHPLC–MS/MS technology, and the amino acids measured in the non-targeted metabolic group were quantitatively verified by MRM method. Through the in-depth study and analysis of the skin of Liaoning cashmere goat, more metabolite information can be obtained, which lays a foundation for improving cashmere quality and economic benefit, and it also provides a new idea for the study of cashmere fiber fineness in the future.

## 2. Materials and Methods

### 2.1. Sample Preparation

The two groups of Liaoning cashmere goats used in the experiment were six coarse cashmere goats and six fine cashmere goats from Liaoning Province Modern Agricultural Production Base Construction Engineering Center. The selected goats were not in the same group, belonged to different paternal lines, had no genetic relationship, their paternal and maternal relationships were more than six generations, and they were not inbred. These goats were 2-year-old ewes with the same feeding management and growth environment. The diameter of coarse cashmere is greater than 17 μm. The diameter of fine cashmere is less than 15 μm. About 1 cm^2^ of skin tissue was taken from the right scapula of Liaoning cashmere goats, which was immediately put into liquid nitrogen and preserved. The experimental Animal Management Committee of Shenyang Agricultural University approved and oversaw all of the experimental procedures utilized in this work (Shenyang, China, 201906099).

### 2.2. Metabolites Extraction

Take the tissue sample of 100 mg prepared by liquid nitrogen grinding, put it in the Eppendorf tube, and add 500 mL of 80% methanol aqueous solution containing 0.1% formic acid. Then, the vortex oscillates while the ice bath remains motionless for five minutes. A portion of the supernatant was collected and diluted with mass spectrometry-grade water, until the methanol concentration was 53% following centrifugation at 15000 rpm for 10 min at 4 °C, and then placed in a centrifuge tube. After repeating the above centrifugation process, the supernatant was collected and injected into LC–MS for analysis. As a QC sample, combine the same volume samples from each experimental sample. 

### 2.3. UHPLC–MS/MS Analysis

#### 2.3.1. Chromatographic Conditions

The chromatograph was Thermo Vanquish UHPLC, performed at a flow rate of 0.2 mL/min and with a linear gradient of 16 min. The sample was added to the hypersil gold column (C18, 100 × 2.1 mm, 1.9 m). The injecting volume was 5 μL in the positive mode and 10 μL in the negative mode. The column temperature was 40 °C. The positive mode eluents were A (0.1% FA in Water) and B (Methanol), and the negative mode eluents were A (5 mM ammonium acetate, pH 9.0) and B (Methanol). The purity of the reagents used was LC–MS, and the brand was Thermo. The solvent gradient was set as follows: 2% B, 1.5 min; 2–100% B, 12 min; 100% B, 14 min; 100–2% B, 14.1 min; 2% B, 17 min.

#### 2.3.2. Mass Spectrometry Conditions

Using Thermo QE mass spectrometer, the scanning range was *m*/*z* 70–1050. The ESI source was configured as follows: Spray Voltage, 3.2 kV; Sheath gas flow rate, 35 arb; Aux Gasflow rate, 10 arb; Capillary Temp, 320 °C; Polarity, positive and negative; Secondary MS/MS scanning is a data-dependent scan.

### 2.4. Identification of Metabolites

Thermo Fisher’s Compound Discoverer 3.1 (CD 3.1) was utilized to process the UHPLC–MS/MS raw data files. The mass–charge ratio, retention time, and other variables were screened. Then, to improve the accuracy of the identification, the peaks of different samples were aligned using a 0.2 min retention time deviation and a 5 ppm mass deviation. For the peak extraction, the target ion is integrated after the peak area has been quantified and the set quality deviation of 5 ppm, 30% signal intensity variation, the minimum signal intensity of 100,000, and adduct ions have been taken into account. The molecular formula is then predicted using the molecular ion peak and fragment ion, and it is compared with the MassList, mzCloud, and mzVault databases. Using a blank sample (53% methanol containing 0.1% formic acid) eliminates the background ions, and the quantitative data are normalized. Finally, the quantitative findings of the data were identified.

### 2.5. Data Analysis

These metabolites were annotated using the KEGG database (http://www.genome.jp/kegg/, accessed on 10 February 2018), HMDB database (http://www.hmdb.ca/, accessed on 27 May 2019), and Lipidmaps database (http://www.lipidmaps.org/, accessed on 9 April 2019). Principal components analysis (PCA) and partial least squares discriminant analysis (PLS-DA) were performed at metaX (a flexible and comprehensive software for processing metabolomics data). We applied univariate analysis (*t*-test) to calculate the statistical significance (*p*-value). The metabolites with VIP > 1, *p*-value < 0.05, and fold change (used to describe the degree of change from an initial value to a final value) ≥ 1.2 or FC ≤ 0.833 were considered to be differential metabolites. Volcano plots were used to filter metabolites of interest based on Log_2_(FC) and −log_10_ (*p*-value) of metabolites.

The correlation between differential metabolites were analyzed by cor () in R language (method = Pearson). Statistically significant correlations between differential metabolites were calculated by cor.mtest () in R language. A *p*-value < 0.05 was considered as statistically significant and correlation plots were plotted by corrplot package in R language. The functions of these metabolites and metabolic pathways were studied using the KEGG database. The metabolic pathways enrichment of differential metabolites was performed; when ratios were satisfied by x/n > y/N, metabolic pathways were considered as an enrichment, and when *p*-value of metabolic pathway < 0.05, metabolic pathways were considered as a statistically significant enrichment.

### 2.6. MRM Validation

The Agilent 1290 Infinity LC ultra-high-performance liquid chromatography system was used to separate the samples. The parameters of the chromatographic column are as follows: Zic HILIC 3.5 µm, 2.1 mm × 150 mm. The mobile phases are A (25 mM ammonium formate + 0.08% FA aqueous solution) and B (0.1% FA acetonitrile). The sample was placed in an automatic sampler at 4 °C, the column temperature was 40 °C, the flow rate was 0.25 mL/min, and the injection volume was 1 μL. The following are the pertinent liquid phase gradients: 90% B, 0 min; 70% B, 12 min; 50% B, 18 min; 40% B, 25 min; 90% B, 30.1 min. At 30.1–37 min, B remains at 90%. In positive mode, the samples were analyzed by 5500 QTRAP mass spectrometer (AB SCIEX). Multiquant software was used to obtain the retention time and chromatographic peak area. The retention time was corrected, and the metabolites were identified using the standard samples of amino acids and their derivatives. Standards of amino acids and their derivatives were used to correct retention time and identify metabolites.

## 3. Results

### 3.1. QC Sample Quality Control

**Figure 1:**
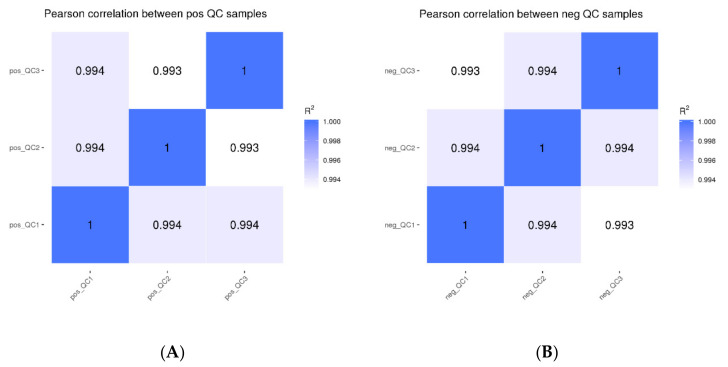
Correlation analysis of QC samples ((**A**): positive mode; (**B**): negative mode).

Based on the peak area value, the Pearson correlation coefficients between QC samples were determined, and the values of R^2^ were all greater than 0.99 (Figure 1). The detection technique overall had strong stability and high data quality, as shown by the high correlation of QC samples, which can be used for further investigation.

### 3.2. Total Sample Principal Component Analysis

**Figure 2:**
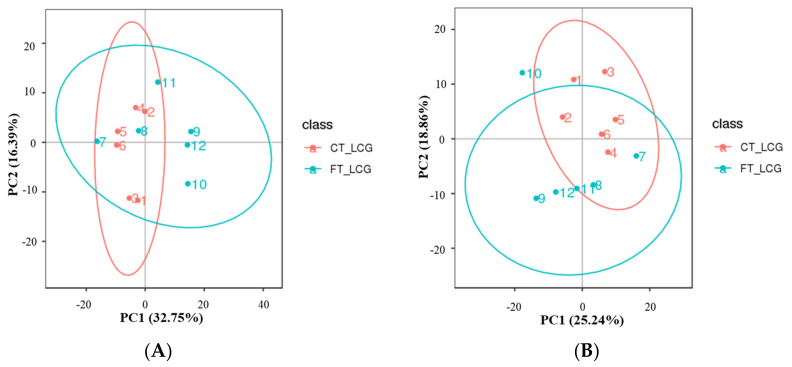
Total sample PCA plot ((**A**): positive mode; (**B**): negative mode). The fractions of the first and second main components are shown in the figure as the abscissa PC1 and ordinate PC2, respectively. The ellipse in the diagram represents the 95 percent confidence interval, and the scattered spots in the figure indicate of experimental samples.

All experimental samples and QC samples had their extracted peaks subjected to PCA analysis. According to the results, QC samples are tightly clustered in positive and negative modes (Figure 2), demonstrating robust experimental repeatability and high data quality [15].

### 3.3. KEGG Pathway Enrichment of all Identified Metabolites 

**Figure 3:**
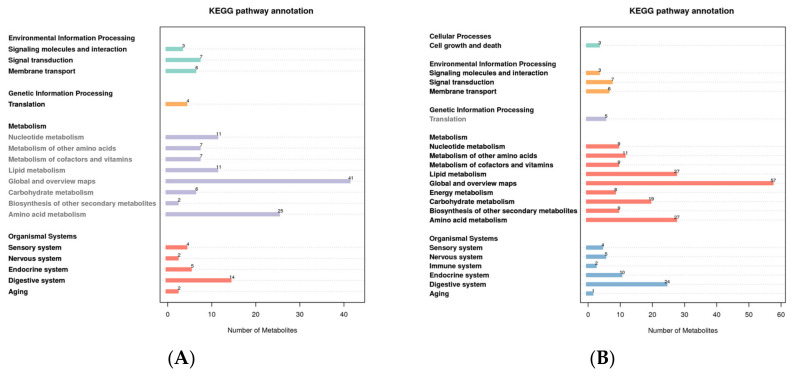
KEGG pathway enrichment of all putatively annotated metabolites ((**A**): positive mode; (**B**): negative mode). The abscissa represents the number of metabolites, and the ordinate represents the KEGG pathway.

For different metabolites to carry out their biological functions in organisms, they always work in concert with one another. The pathway-based analysis aids in a deeper understanding of these bodily functions. In the positive mode, the metabolites were primarily enriched in global and overview maps and amino acid metabolism, according to KEGG pathway analysis of all the putatively annotated metabolites. Metabolites were primarily enriched on global and overview maps, amino acid metabolism, and lipid metabolism in the negative mode (Figure 3).

### 3.4. HMDB Classification Notes

**Figure 4:**
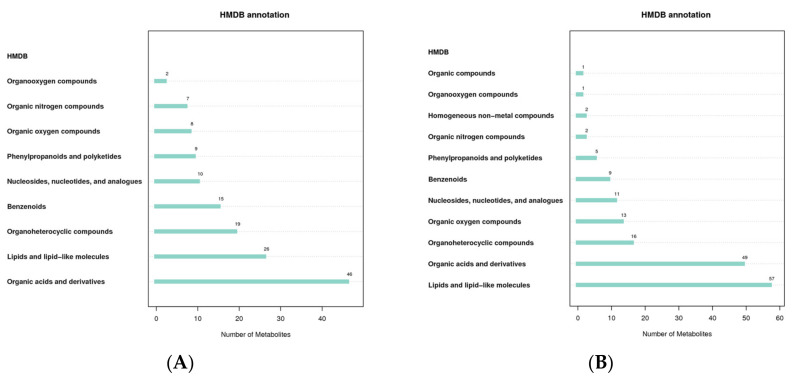
HMDB classification notes ((**A**): positive mode; (**B**): negative mode). The abscissa represents the number of metabolites, and the ordinate represents the HMDB entry annotated.

The Human Metabolome Database (HMDB) contains comprehensive data on the small molecule metabolites found in the human body, as well as information on their biological functions, physiological concentrations, disease associations, chemical reactions, metabolic pathways, and other factors. According to the classification and attribution of the identified metabolites, it was found that the metabolites were mainly concentrated in organic acids and derivatives and lipids and lipid-like molecules in both positive and negative modes (Figure 4).

### 3.5. LIPID MAPS Classification Notes

**Figure 5:**
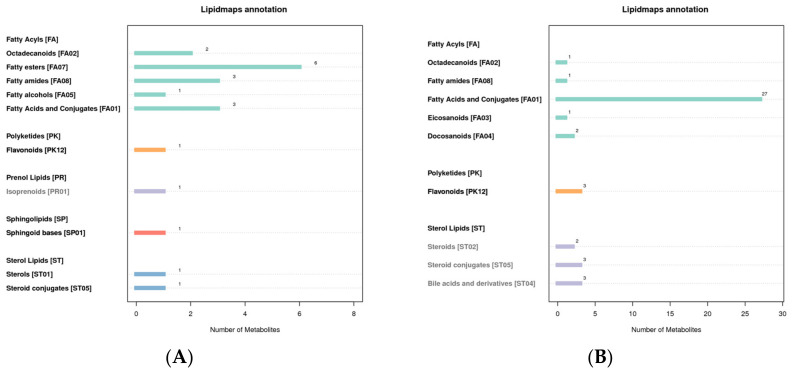
LIPID MAPS classification notes ((**A**): positive mode; (**B**): negative mode). The abscissa represents the number of metabolites, and the ordinate represents the LIPID MAPS entry annotated.

The LIPID MAPS database can annotate the eight lipids’ categories, their subcategories, and the identified metabolites. The results show that the lipids in the metabolites in the positive mode are mainly fatty ester, and the lipids in the negative mode are mainly fatty acids and conjugates (Figure 5).

### 3.6. Partial Least Squares Discrimination Analysis (PLS-DA)

**Figure 6:**
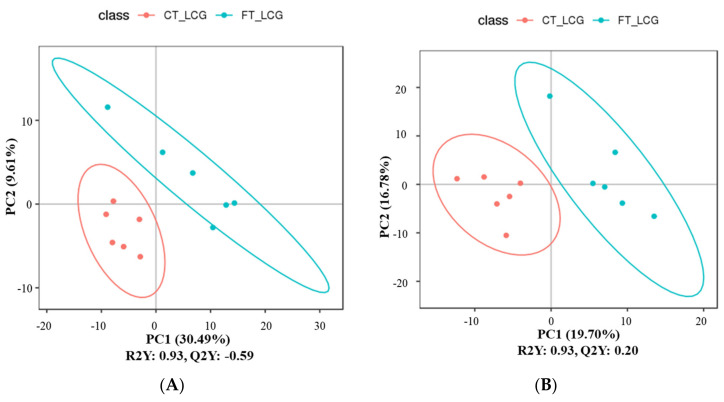
PLS-DA score scatter plots ((**A**): positive mode; (**B**): negative mode). The sample’s score on the first principal component is represented by the abscissa, while the ordinate represents the sample’s score on the second principal component. R2Y is the model’s interpretation rate, and Q2Y is used to assess the PLS-DA model’s predictive power.

**Figure 7:**
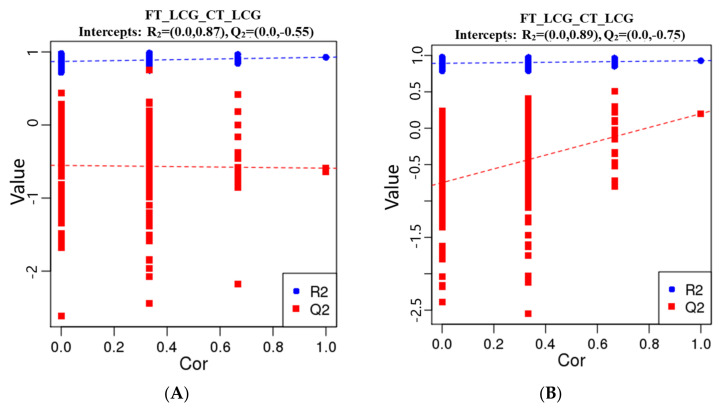
PLS-DA sorting verification diagram ((**A**): positive mode; (**B**): negative mode). The scores for R2 and Q2 make up the ordinate, while the abscissa represents the correlation between the original grouping Y and the random grouping Y.

The metabolites were characterized by PLS-DA (Figure 6), which showed significant differences between CT_LCG and FT_LCG in the positive and negative modes. Each group of samples was basically within a 95% confidence interval. In order to judge the quality of the model, we conducted a permutation test on the model to check whether the model is “over-fitted”. We randomly disrupt the grouping marks of each sample and then model and predict them. Each modeling corresponds to a set of values of R2 and Q2. According to the values of Q2 and R2 after 200 disruptions and modeling, their regression lines can be obtained [16], as shown in Figure 7. The results of sequencing verification in positive and negative modes show that R2 is larger than Q2, and the intercept between the Q2 regression line and the Y–axis is less than 0, indicating that the model does not “overfit” and is statistically reliable (Figure 7).

### 3.7. Differential Metabolite Analysis

**Figure 8:**
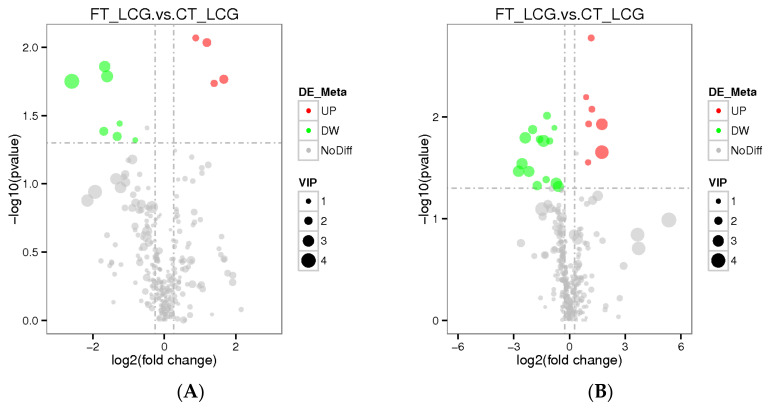
Differential metabolite volcano plots ((**A**): positive mode; (**B**): negative mode). The abscissa represents the multiple changes in expression of metabolites in different groups (log_2_FC), and the ordinate represents the significant level of difference (−log_10_ *p*-value). Each dot on the volcano plot stands for a different metabolite; the red dot indicates a metabolite that has been significantly up-regulated, while the green dot indicates a metabolite that has been significantly down-regulated, and the size of the dot denotes the VIP value.

The total number of metabolites identified in positive and negative modes was 286 and 339, respectively. The VIP value indicates the contribution rate of different metabolites in different groups. The screening criteria of differential metabolites are set as VIP > 1.0, FC > 1.2 or FC < 0.833, and *p*-value < 0.05. The differential metabolites were screened out. According to the findings, four metabolites were significantly up-regulated under positive mode, while seven metabolites were significantly down-regulated. Seven metabolites were significantly up-regulated under negative mode, whereas fourteen were significantly down-regulated (Figure 8). Among them, Gly–Phe in positive mode and taurochenodeoxycholate in negative mode were up-regulated and the difference was the most significant (Table 1 and Table 2).

### 3.8. Correlation Analysis of Differential Metabolites

**Figure 9:**
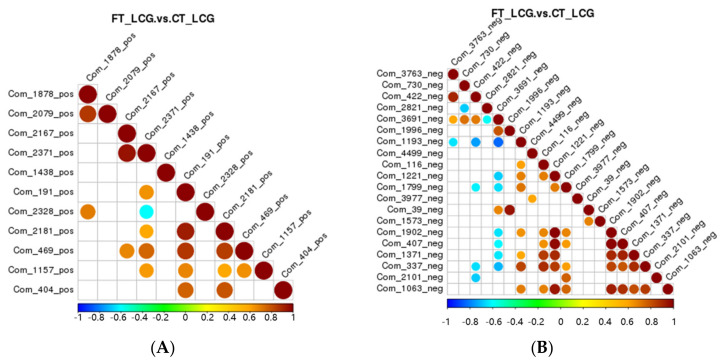
Differential metabolite correlation diagram ((**A**): positive mode; (**B**): negative mode). Red denotes positive correlation, and the highest correlation is 1. Blue means negative correlation, and the lowest correlation is −1. *p* > 0.05 is true of the portion without color. The top 20 differential metabolites are correlated in the figure in order of *p*-value from minimum to maximum.

Correlation analysis can be used to quantify the metabolic closeness of metabolites with notable differences and to understand how metabolites are regulated in concert due to changes in biological state. The correlation between metabolites with notable changes was examined by measuring the Pearson correlation coefficient between all metabolites. Celestolide and neodiosmin have a negative association in the positive mode, but celestolide and other metabolites correlate positively. Taurochenodeoxyacetate has a negative correlation with delta-tridecalactone and a positive correlation with other metabolites in the negative mode (Figure 9).

### 3.9. KEGG Pathway Enrichment of the Differential Metabolites 

**Figure 10:**
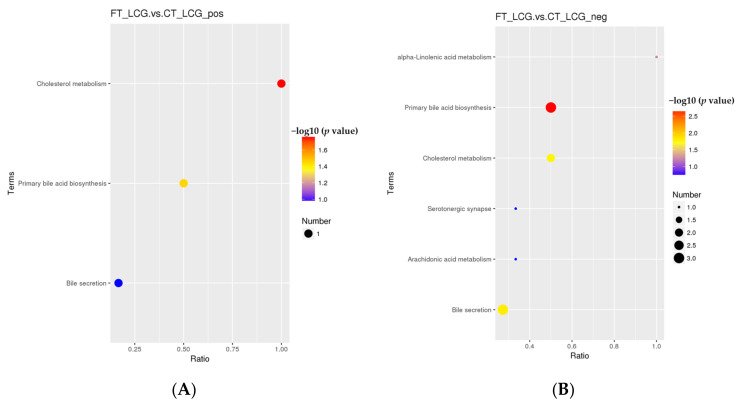
KEGG enrichment bubble diagram ((**A**): positive mode; (**B**): negative mode). The abscissa is x/y, and the value increases with the pathway’s differential metabolite enrichment level. The hypergeometric test’s *p*-value is represented by the color of the point, and the smaller the value, the higher the test’s reliability. The number of distinct metabolites in the associated route is indicated by the size of the point.

According to the KEGG enrichment analysis of the selected significant differential metabolites, only the three pathways of cholesterol metabolism, primary bile acid biosynthesis, and bile secretion were enriched in the positive mode (Figure 10). Only one significant difference metabolite, glycochenodeoxycholic acid, was enriched (Table 3). Six KEGG pathways were enriched in the negative mode, which are taurochenodeoxycholate, cholic acid, taurochenodeoxycholic acid, jasmonic acid, and prostaglandin B2 (Figure 10). Among them, five metabolites with significant differences were enriched, namely, taurochenodeoxycholate, cholic acid, taurochenodeoxycholic acid, jasmonic acid, and prostaglandin B2 (Table 4). There were significant differences in primary bile acid biosynthesis and bile secretion in positive and negative modes. 

### 3.10. Validation Experiment

**Figure 11:**
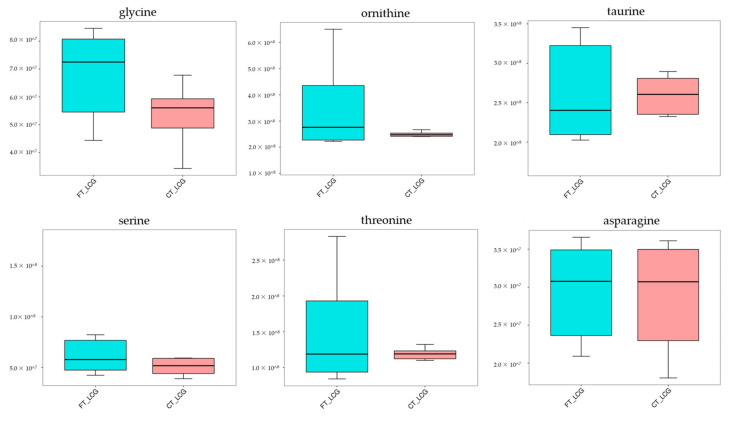
Trend map of relative expression of metabolites.

**Figure 12:**
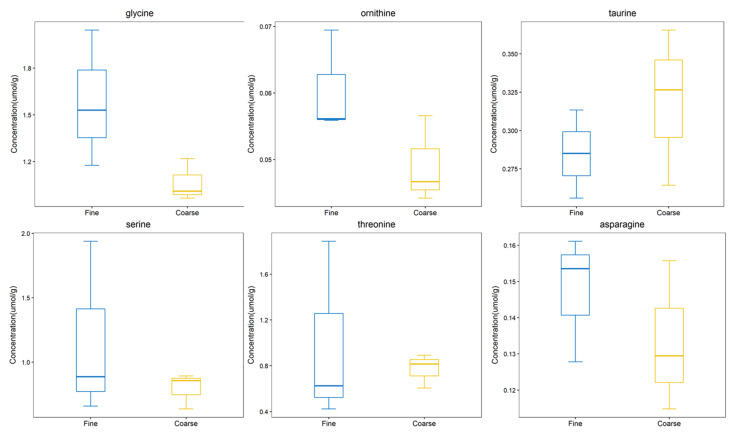
Trend map of metabolite expression.

From the comprehensive analysis of KEGG metabolic pathway and its metabolic mechanism, it was found that the *BAAT* gene was annotated in the primary bile acid biosynthesis pathway, and previous studies have shown that this gene may be related to cashmere performance [17]. Moreover, the expression of the *BAAT* gene directly acts on taurochenodeoxycholate, glycochenodeoxycholic acid, Taurocholate, and Glycocholate. At the same time, it interacts with taurine and glycine. Amino acids are the smallest molecules of all metabolites. In addition to serving as the building blocks for the creation of proteins, amino acids are a crucial source of energy for cells. Amino acids also play an essential role as signal molecules in intracellular signal transduction and metabolic regulation [18]. There are more sulfur-containing amino acids in hair. In addition, serine, glycine, arginine, aspartic acid, alanine, ornithine, threonine, phenylalanine, glutamic acid, and lysine are the primary amino acids in hair [19,20]. Taurine, serine, glycine, ornithine, threonine, and asparagine were detected in CT_LCG and FT_LCG, and the relative expression is relatively high (Figure 11). We verified the six amino acids by MRM and found that the changing trend of these amino acids was consistent with that based on UHPLC–MS/MS technology (Figure 12). It can be seen that glycine, ornithine, and asparagine play a positive role in regulating cashmere.

## 4. Discussion

In this study, we used UHPLC–MS/MS technology to detect differences in the skin of coarse and fine cashmere LCG. Metabolites in the two groups of samples varied, as was to be expected. Some studies have shown that melatonin can stimulate the development of secondary hair follicles in cashmere goats, increase cashmere production, and reduce fiber diameter [21]. The changes in serum melatonin, insulin-like growth factor, and prolactin concentrations in LCG during the rapid cashmere growth period are related to cashmere growth. Tryptophan can promote cashmere growth by increasing diurnal insulin-like growth factor secretion and nocturnal melatonin [22]. We detected 32 differential metabolites in the skin of LCG, of which 11 were up-regulated and 21 down-regulated. Further investigation reveals that these metabolites could be crucial to the fineness of cashmere.

Hair abounds with sulfur amino acids, including cystine, cysteine, and methionine [23]. Additionally, the sulfur level directly impacts the characteristics of wool fiber, and amino acids containing sulfur are crucial for animal metabolism and function. Sherlock et al. showed that supplementing sulfur-containing amino acids could significantly increase wool yield and sulfur concentration in wool [24]. The nutritional supply of sulfur-containing amino acids can significantly increase the mitosis rate of wool hair follicle globular cells [25]. The study by Prusiewicz-Witaszek et al. showed that adding methionine and lysine to rabbit feed could affect the synthesis of keratin in hair and increase the yield of villi [26]. A total of two sulfur-containing differential metabolites, taurochenodeoxycholate and taurochenodeoxycholic acid, were detected in this study (Table 2). These two metabolites are up-regulated and contribute to the regulation of cashmere’s fineness. The metabolism of methionine and the cysteine production of taurine is a naturally occurring β-amino acid. Taurine has been found to be absorbed by connective tissue sheath, proximal outer root sheath, and hair bulb, promoting hair survival in vitro and preventing harmful effects on hair follicles induced by TGF-β1 [27]. Tauroursodeoxycholic acid can inhibit the proliferation of keratinocytes in a dose-dependent manner and has a reversible inhibitory effect on the growth of human keratinocytes [28]. Taurine has great potential for anti-alopecia, especially for chemical stress-induced alopecia [29]. *KAP8.1*, the structural gene responsible for cashmere, contains high levels of glycine and tyrosine [30]. The contents of tyrosine, glycine, leucine, and phenylalanine in ortho cortical cells of wool are high [31]. The heads and/or tails of epidermal keratins are glycine and phenylalanine abundant, but alanine is lacking [32]. In this study, there is a difference in taurine and glycine expression between FT_LCG and CT_LCG (Figure 12), which indicates that these two amino acids may be involved in the regulation of cashmere fineness.

At present, studies have shown that Wnt, Shh, TGF-b, Notch, NF-kappa B, and PPAR signal pathways may be related to cashmere development [33,34,35,36,37]. In this study, we enriched six KEGG pathways in positive and negative modes, among which cholesterol metabolism, primary bile acid biosynthesis, and bile secretion were enriched in both ion modes (Table 3, 4). The three pathways were annotated with four highly significant differences in metabolites: taurochenodeoxycholate, cholic acid, taurochenodeoxycholic acid, and glycochenodeoxycholic acid. Lactosylceramidase I in skin fibroblasts can be stimulated by pure taurocholate, while lactosylceramidase II can be stimulated by taurocholate or pure sugar deoxycholate, taurine deoxycholate, and taurine goose deoxycholate to catalyze the hydrolysis of lactose ceramide [33]. Cholesterol is crucial to the physiology of the skin and is a component of epidermal barrier function [38]. For a long time, cholesterol has been considered as an important factor affecting hair growth and plays an important role in hair follicle biology [39]. Cholesterol modification is also necessary for the signal transduction of Wnt/β-catenin and Hedgehog pathways [40]; these pathways are the basis for controlling the circulation of hair follicles [41]. Although no literature has reported that cholesterol metabolism, primary bile acid biosynthesis, and the bile secretion signal pathway are related to cashmere fineness, there are related metabolites in these pathways that are closely related to hair follicle development, so it can be inferred that these pathways may regulate cashmere growth and fineness, but this needs further study to verify.

## 5. Conclusions

To summarize, this study analyzed the metabolomics of coarse and fine cashmere skin of Liaoning cashmere goats; 32 differential metabolites were screened and enriched into six KEGG pathways. The findings suggest that Gly–Phe and taurochenodeoxycholate may be crucial in regulating cashmere’s growth, development, and fineness. This study offers a theoretical foundation for further investigating the variables influencing cashmere’s fineness.

## Figures and Tables

**Table 1:** Significant difference metabolites in positive mode.

ID	Name	Formula	FC	log_2_FC	*p* Value	VIP	Trend
Com_1878_pos	Gly–Phe	C_11_H_14_N_2_O_3_	1.840516	0.88011	0.008541	1.355301	up
Com_2079_pos	Tyrosylalanine	C_12_H_16_N_2_O_4_	2.28476	1.192043	0.009232	2.011242	up
Com_2167_pos	6,7,8-trimethoxy-2-(2-phenoxy-3-pyridyl)-4H-3,1-benzoxazin-4-one	C_22_H_18_N_2_O_6_	0.314242	−1.67005	0.013825	2.865879	down
Com_2371_pos	Neodiosmin	C_28_H_32_O_15_	0.329646	−1.60101	0.016317	3.036509	down
Com_1438_pos	Glycochenodeoxycholic acid	C_26_H_43_NO_5_	3.171151	1.665007	0.017141	2.102527	up
Com_191_pos	4-(2,3-dihydro-1,4-benzodioxin-6-yl)butanoic acid	C_12_H_14_O_4_	0.165891	−2.59169	0.017738	4.05138	down
Com_2328_pos	Celestolide	C_17_H_24_O	2.629355	1.394709	0.018376	1.504881	up
Com_2181_pos	3-hydroxy-*N*-(1-hydroxy-4-methylpentan-2-yl)-5-oxo-6-phenylhexanamide	C_18_H_27_NO_4_	0.420322	−1.25043	0.036147	1.137918	down
Com_469_pos	3-Methylcrotonylglycine	C_7_H_11_NO_3_	0.308891	−1.69483	0.041219	2.062733	down
Com_1157_pos	ACar 10:1	C_17_H_32_NO_4_	0.400311	−1.32081	0.044941	2.120841	down
Com_404_pos	indoline-2-carboxylic acid	C_9_H_9_NO_2_	0.568419	−0.81497	0.04785	1.004339	down

**Table 2:** Significant difference metabolites in negative mode.

ID	Name	Formula	FC	log_2_FC	*p* Value	VIP	Trend
Com_3763_neg	Taurochenodeoxycholate	C_26_H_44_N NaO_6_S	2.245599	1.167101	0.001669	1.407966	up
Com_730_neg	13,14-Dihydro-15-keto Prostaglandin A2	C_20_H_30_O_4_	1.856423	0.892526	0.006386	1.124458	up
Com_422_neg	Taurochenodeoxycholic acid	C_26_H_45_NO_6_S	2.302059	1.202925	0.008387	1.380152	up
Com_2821_neg	PE (18:1e/18:1)	C_41_H_80_NO_7_P	0.431353	−1.21306	0.009712	1.747769	down
Com_3691_neg	Prostaglandin B2	C_20_H_30_O_4_	2.039076	1.027916	0.011712	1.406344	up
Com_1996_neg	7-Ketodeoxycholic acid	C_24_H_38_O_5_	3.34657	1.742683	0.011779	3.023359	up
Com_1193_neg	Delta-Tridecalactone	C_13_H_24_O_2_	0.568493	−0.81479	0.012783	1.015299	down
Com_4499_neg	PE (18:2e/18:2)	C_41_H_76_NO_7_P	0.25246	−1.98587	0.013294	2.21477	down
Com_116_neg	*N*-Isobutyrylglycine	C_6_H_11_NO_3_	0.19186	−2.38187	0.016014	2.980363	down
Com_1221_neg	*N*-Acetylglycine	C_4_H_7_NO_3_	0.326927	−1.61296	0.016573	1.708988	down
Com_1799_neg	Jasmonic acid	C_12_H_18_O_3_	0.378752	−1.40068	0.017172	3.143533	down
Com_3977_neg	(3 shan,5 fou,9 fou)-3,23-Dihydroxy-1-oxoolean-12-en-28-oic acid	C_30_H_46_O_5_	0.478919	−1.06215	0.017208	1.489396	down
Com_39_neg	Cholic acid	C_24_H_40_O_5_	3.341702	1.740583	0.022171	3.659888	up
Com_1573_neg	*N*-Acetylsphingosine	C_20_H_39_NO_3_	2.000667	1.000481	0.027948	1.19617	up
Com_1902_neg	5-(3-cyclohexylprop-1-ynyl)nicotinic acid	C_15_H_17_NO_2_	0.169535	−2.56034	0.028812	2.900065	down
Com_407_neg	Hexanoylglycine	C_8_H_15_NO_3_	0.149356	−2.74317	0.034156	2.920229	down
Com_1371_neg	Capryloylglycine	C_10_H_19_NO_3_	0.220021	−2.18429	0.034311	2.813614	down
Com_337_neg	*N*-Tigloylglycine	C_7_H_11_NO_3_	0.417917	−1.25871	0.041284	1.575588	down
Com_2101_neg	Isophorone	C_9_H_14_O	0.609557	−0.71417	0.044981	2.975498	down
Com_1063_neg	3-[(methoxycarbonyl)amino]-2,2,3-trimethylbutanoic acid	C_9_H_17_NO_4_	0.299	−1.74178	0.047286	2.261636	down
Com_326_neg	3,3-Dimethylglutaric acid	C_7_H_12_O_4_	0.658063	−0.6037	0.048218	2.792619	down

**Table 3:** KEGG enrichment pathway in positive mode.

Map ID	Map Title	*p* Value	x	y	Enrich Direct	Meta IDs	Name
map04979	Cholesterol metabolism	0.017544	1	1	Over	Com_1438_pos	Glycochenodeoxycholic acid
map00120	Primary bile acid biosynthesis	0.035088	1	2	Over	Com_1438_pos	Glycochenodeoxycholic acid
map04976	Bile secretion	0.105263	1	6	Over	Com_1438_pos	Glycochenodeoxycholic acid

Notes: The x denotes the number of differential metabolites associated with the pathway; y denotes the number of background (all) metabolites related to the pathway.

**Table 4:** KEGG enrichment pathway in negative mode.

MapID	MapTitle	*p* Value	x	y	EnrichDirect	MetaIDs	Name
map00120	Primary bile acid biosynthesis	0.002056	3	6	Over	Com_3763_neg Com_39_neg Com_422_neg	Taurochenodeoxycholate; Cholic acid; Taurochenodeoxycholic acid
map04976	Bile secretion	0.01536	3	11	Over	Com_3763_neg Com_39_neg Com_422_neg	Taurochenodeoxycholate; Cholic acid;Taurochenodeoxycholic acid
map04979	Cholesterol metabolism	0.016769	2	4	Over	Com_3763_neg Com_422_neg	Taurochenodeoxycholate; Taurochenodeoxycholic acid
map00592	alpha-Linolenic acid metabolism	0.060241	1	1	Over	Com_1799_neg	Jasmonic acid
map00590	Arachidonic acid metabolism	0.172016	1	3	Over	Com_3691_neg	Prostaglandin B2
map04726	Serotonergic synapse	0.172016	1	3	Over	Com_3691_neg	Prostaglandin B2

Notes: The x represents the number of differential metabolites associated with the pathway; y represents the number of background (all) metabolites related to the pathway.

## Data Availability

Not applicable.

## Sample Availability

Samples of the compounds are available from the authors.

## References

[1] Wang S., Ge W., Luo Z., Guo Y., Jiao B., Qu L., Zhang Z., Wang X. (2017). Integrated analysis of coding genes and non-coding RNAs during hair follicle cycle of cashmere goat (Capra hircus). BMC Genom..

[2] Liu X., Jiang H.Z., Song X.C. (2020). Analysis of annual effect of cashmere performance of Liaoning cashmere goats. Anim. Husb. Vet. Med..

[3] Dunn W.B., Ellis D. (2005). Metabolomics: Current analytical platforms and methodologies. TrAC Trends Anal. Chem..

[4] Fiehn O. (2002). Metabolomics—The link between genotypes and phenotypes. Plant Mol. Biol..

[5] Delplancke T.D., De Seymour J.V., Tong C., Sulek K., Xia Y., Zhang H., Han T.L., Baker P.N. (2018). Analysis of sequential hair segments reflects changes in the metabolome across the trimesters of pregnancy. Sci. Rep..

[6] Wang X., Cai C., Liang Q., Xia M., Lai L., Wu X., Jiang X., Cheng H., Song Y., Zhou Q. (2022). Integrated Transcriptomics and Metabolomics Analyses of Stress-Induced Murine Hair Follicle Growth Inhibition. Front. Mol. Biosci..

[7] Elpa D.P., Chiu H.Y., Wu S.P., Urban P.L. (2021). Skin Metabolomics. Trends Endocrinol. Metab..

[8] Dutkiewicz E.P., Hsieh K.-T., Urban P.L., Chiu H.-Y. (2020). Temporal Correlations of Skin and Blood Metabolites with Clinical Outcomes of Biologic Therapy in Psoriasis. J. Appl. Lab. Med..

[9] Pohla L., Ottas A., Kaldvee B., Abram K., Soomets U., Zilmer M., Reemann P., Jaks V., Kingo K. (2020). Hyperproliferation is the main driver of metabolomic changes in psoriasis lesional skin. Sci. Rep..

[10] Caboni P., Murgia A., Porcu A., Manis C., Ibba I., Contu M., Scano P. (2019). A metabolomics comparison between sheeps and goats milk. Food Res. Int..

[11] Liu H., Guo X., Zhao Q., Qin Y., Zhang J. (2019). Lipidomics analysis for identifying the geographical origin and lactation stage of goat milk. Food Chem..

[12] Scano P., Murgia A., Pirisi F.M., Caboni P. (2014). A gas chromatography-mass spectrometry-based metabolomic approach for the characterization of goat milk compared with cow milk. J. Dairy Sci..

[13] Sun L., Guo Y., Fan Y., Nie H., Wang R., Wang F. (2017). Metabolic profiling of stages of healthy pregnancy in Hu sheep using nuclear magnetic resonance (NMR). Theriogenology.

[14] Jang W.-J., Choi J.Y., Park B., Seo J.H., Seo Y.H., Lee S., Jeong C.-H., Lee S. (2019). Hair Metabolomics in Animal Studies and Clinical Settings. Molecules.

[15] Sumner L.W., Amberg A., Barrett D., Beale M.H., Beger R., Daykin C.A., Fan T.W., Fiehn O., Goodacre R., Griffin J.L. (2007). Proposed minimum reporting standards for chemical analysis Chemical Analysis Working Group (CAWG) Metabolomics Standards Initiative (MSI). Metabolomics.

[16] Wang J., Pu S., Sun Y., Li Z., Niu M., Yan X., Zhao Y., Wang L., Qin X., Ma Z. (2014). Metabolomic Profiling of Autoimmune Hepatitis: The Diagnostic Utility of Nuclear Magnetic Resonance Spectroscopy. J. Proteome Res..

[17] Cai W., Xu Y., Bai Z., Lin G., Wang L., Dou X., Han D., Wang Z., Wang J., Zhang X. (2022). Association analysis for SNPs of BAAT and COL1A1 genes with cashmere production performance and other production traits in Liaoning cashmere goats. Anim. Biotechnol..

[18] Bröer S., Bröer A. (2017). Amino acid homeostasis and signalling in mammalian cells and organisms. Biochem. J..

[19] Sande V.M. (1970). Hair amino acids: Normal values and results in metabolic errors. Arch. Dis. Child..

[20] Yoshino M., Kubota K., Yoshida I., Murakami T., Yamashita F. (1982). Argininemia: Report of a New Case and Mechanisms of Orotic Aciduria and Hyperammonemia. Adv. Exp. Med. Biol..

[21] Yang C.H., Xu J.H., Ren Q.C., Duan T., Mo F., Zhang W. (2019). Melatonin promotes secondary hair follicle development of early postnatal cashmere goat and improves cashmere quantity and quality by enhancing antioxidant capacity and suppressing apoptosis. J. Pineal. Res..

[22] Ma H., Zhang W., Song W.H., Sun P., Jia Z.H. (2012). Effects of tryptophan supplementation on cashmere fiber characteristics, serum tryptophan, and related hormone concentrations in cashmere goats. Domest. Anim. Endocrinol..

[23] Zahn H., Gattner H.-G. (1997). Hair sulfur amino acid analysis. Exs.

[24] Sherlock R.G., Harris P.M., Lee J., Wickham G.A., Woods J.L., McCutcheon S.N. (2001). Intake and long-term cysteine supplementation change wool characteristics of Romney sheep. Aust. J. Agric. Res..

[25] Hynd P.I., Rogers G.E., Reis P.J., Ward K.A., Marshall R.C. (1988). Factors Influencing Cellular Events in the Wool Follicle. The Biology of Wool and Hair.

[26] Prusiewicz-Witaszek U. (1975). Changes in the biosynthesis of keratin in the hair following supplementing rabbits’ basic feed with methionine and lysine. Polskie Arch. Weter..

[27] Collin C., Gautier B., Gaillard O., Hallegot P., Chabane S., Bastien P., Peyron M., Bouleau M., Thibaut S., Pruche F. (2006). Protective effects of taurine on human hair follicle grown in vitro. Int. J. Cosmet. Sci..

[28] Yamaguchi Y., Itami S., Nishida K., Ando Y., Okamoto S., Hosokawa K., Yoshikawa K. (1998). Taurin-conjugated ursodeoxycholic acid has a reversible inhibitory effect on human keratinocyte growth. J. Dermatol. Sci..

[29] Kim H., Chang H., Lee N.-H. (2013). Simulative Evaluation of Taurine Against Alopecia Caused by Stress in Caenorhabditis Elegans.

[30] Zhao M., Chen H., Wang X., Yu H., Wang M., Wang J., Lan X.Y., Zhang C.F., Zhang L.Z., Guo Y.K. (2009). aPCR-SSCP and DNA sequencing detecting two silent SNPs at KAP8.1 gene in the cashmere goat. Mol. Biol. Rep..

[31] Chapman G.V., Bradbury J.H. (1968). The chemical composition of wool. 7. Separation and analysis of orthocortex and paracortex. Arch. Biochem. Biophys.

[32] Strnad P., Usachov V., Debes C., Gräter F., Parry D.A., Omary M.B. (2011). Unique amino acid signatures that are evolutionarily conserved distinguish simple-type, epidermal and hair keratins. J. Cell Sci..

[33] Dai B., Liang H., Guo D.-D., Bi Z.-W., Yuan J.-L., Jin Y., Huan L., Guo X.-D., Cang M., Liu D.J. (2019). The Overexpression of Tβ4 in the Hair Follicle Tissue of Alpas Cashmere Goats Increases Cashmere Yield and Promotes Hair Follicle Development. Animals.

[34] Geng R., Yuan C., Chen Y. (2013). Exploring Differentially Expressed Genes by RNA-Seq in Cashmere Goat (Capra hircus) Skin during Hair Follicle Development and Cycling. PLoS ONE.

[35] Kloepper J.E., Ernst N., Krieger K., Bodó E., Bíró T., Haslam I.S., Schmidt-Ullrich R., Paus R. (2014). NF-κB activity is required for anagen maintenance in human hair follicles in vitro. J. Investig. Dermatol..

[36] Laurikkala J., Pispa J., Jung H.-S., Nieminen P., Mikkola M., Wang X., Saarialho-Kere U., Galceran J., Grosschedl R., Thesleff I. (2002). Regulation of hair follicle development by the TNF signal ectodysplasin and its receptor Edar. Development.

[37] Yue Y., Guo T., Yuan C., Liu J., Guo J., Feng R., Niu C., Sun X., Yang B. (2016). Integrated Analysis of the Roles of Long Noncoding RNA and Coding RNA Expression in Sheep (Ovis aries) Skin during Initiation of Secondary Hair Follicle. PLoS ONE.

[38] Feingold K.R. (2009). The outer frontier: The importance of lipid metabolism in the skin. J. Lipid Res..

[39] Palmer M., Blakeborough L., Harries M., Haslam I.S. (2020). Cholesterol homeostasis: Links to hair follicle biology and hair disorders. Exp. Dermatol..

[40] Incardona J.P., Eaton S. (2000). Cholesterol in signal transduction. Curr. Opin. Cell Biol..

[41] Lee J., Tumbar T. (2012). Hairy tale of signaling in hair follicle development and cycling. Semin. Cell Dev. Biol..

